# Supplementary material for: Sequence and structural alignments reveal insights into ANKLE2 evolution and function
Source: Protein Sci. 2026 May 15;35(6):e70618. doi: 10.1002/pro.70618 (PMC13179284; doi:10.1002/pro.70618)
Supplement: Supplementary file 1 — Figure S1. Representative confocal microscopy images of ANKLE2 orthologs. Figure S2. Sequence identity versus structural similarity matrix for full‐length ANKLE2 orthologs. Figure S3. Foldseek and FATCAT/US‐align as tools to evaluate similarity of individual ANKLE2 domains. Figure S4. Sequence‐structure matrix for uncharacterized domain #1. Figure S5. Sequence alignments of GIY‐YIG regions of SLX1A, ANKLE1, and ANKLE2. Figure S6. Protein production and purification validation. Figure S7. Sequence‐structure matrix for uncharacterized domain #2. Figure S8. Foldseek results for ANKLE2 uncharacterized domain #2. Figure S9. ANKLE2 does not bind with dsDNA by electrophoretic mobility shift assay. Figure S10. Observations of micronuclei during etoposide treatment. Table S2. Sequence accession numbers for all protein sequences used. Table S3. Antibody usage details for all antibodies used. [file PRO-35-e70618-s001.docx]

**Supplementary Material**


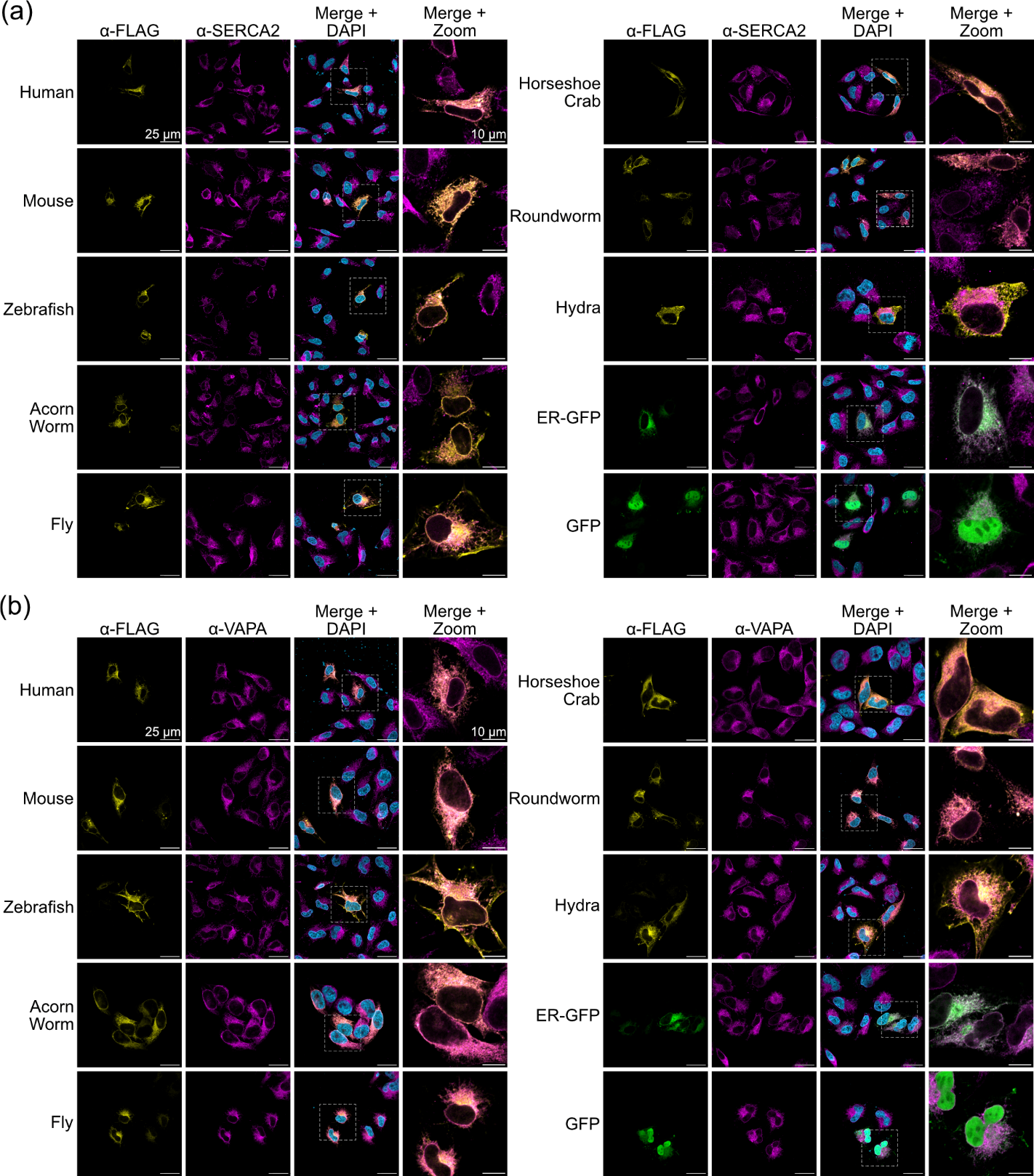


**Figure S1: Representative confocal microscopy images of ANKLE2 orthologs.**  (a-b) HeLa cells were plated on glass cover slips overnight and transfected with ANKLE2-3xFLAG orthologs or GFP-3xFLAG controls. Cells were then fixed in paraformaldehyde prior to immunostaining for FLAG (yellow/green), SERCA2 (a, magenta) or VAPA (b, magenta). Nuclei were visualized with 1:10000 Hoechst (cyan). Cover slips were imaged at 63X using confocal microscopy. Final image colors were altered from the wavelength they were taken for visual clarity and consistency. For GFP samples, FLAG and GFP signal could not be distinguished and therefore remain visualized in the green channel.


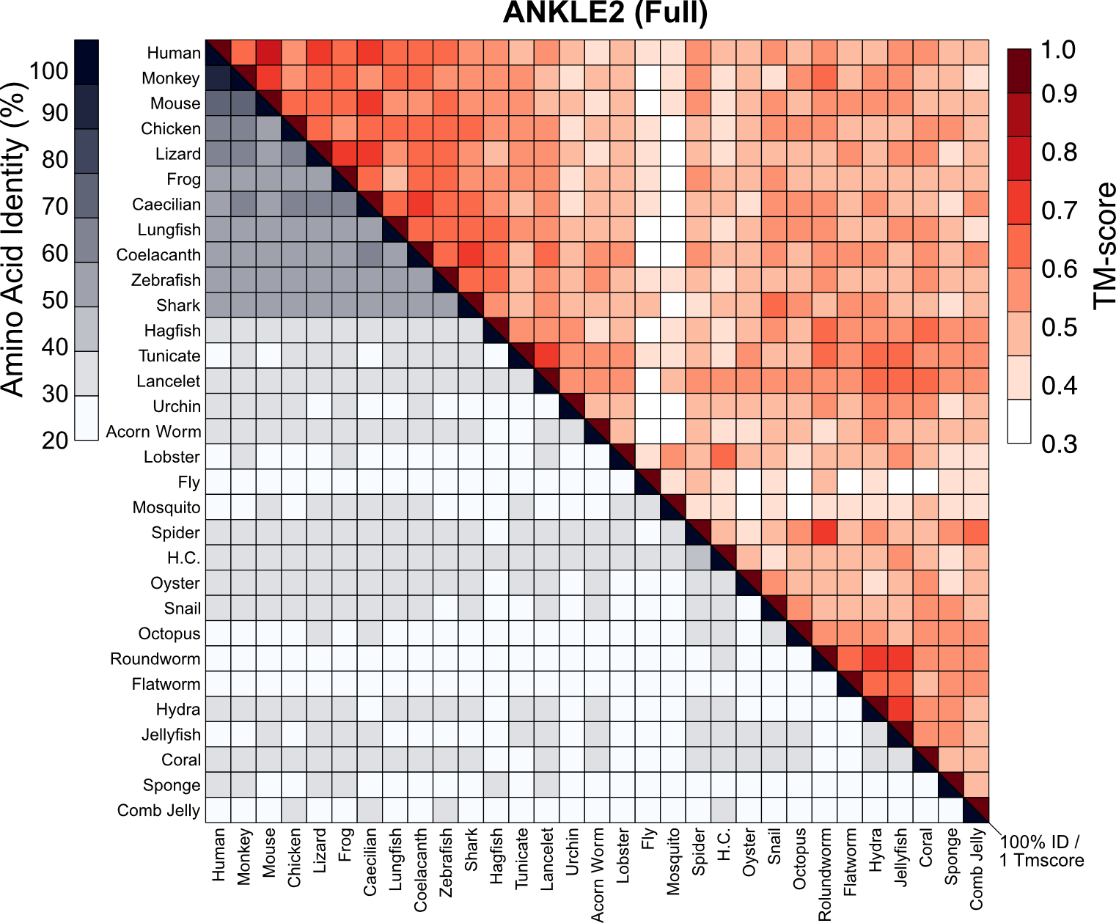


**Figure S2: Sequence identity versus structural similarity matrix for full-length ANKLE2 orthologs.**  Amino acid sequence identities of ANKLE2 orthologs were measured using ClustalOmega. AlphaFold2 structural predictions were acquired or generated and compared against each other using US-align.


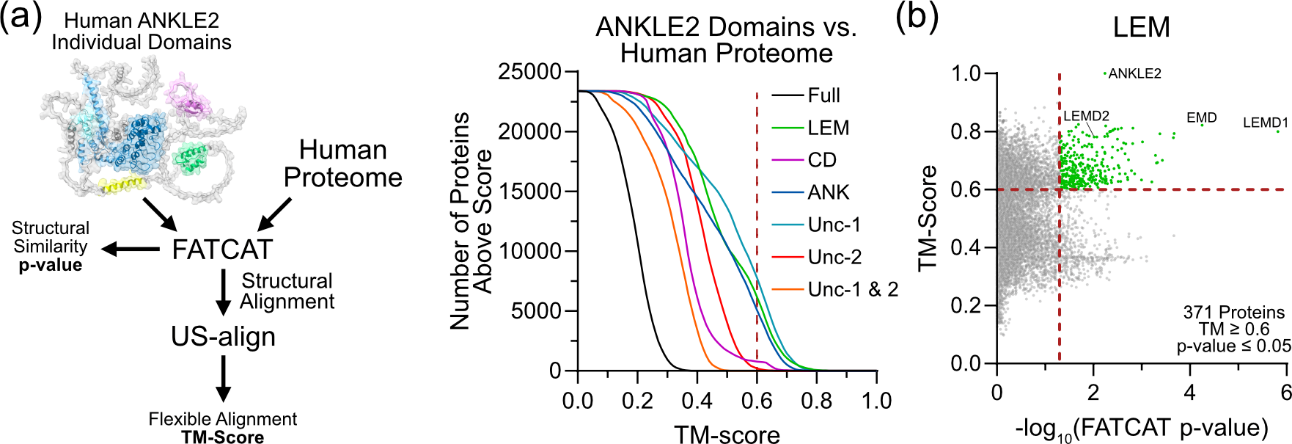


**Figure S3:** **Foldseek and FATCAT/US-align as tools to evaluate similarity of individual ANKLE2 domains.**  (a) Workflow for comparing individual ANKLE2 domains against the human proteome using FATCAT🡪US-align flexible protein alignments. Waterfall plot shows the number of proteins above each TM-score. (b) Flexible alignments of the ANKLE2-LEM domain reveal other known LEM proteins among highest scoring hits.


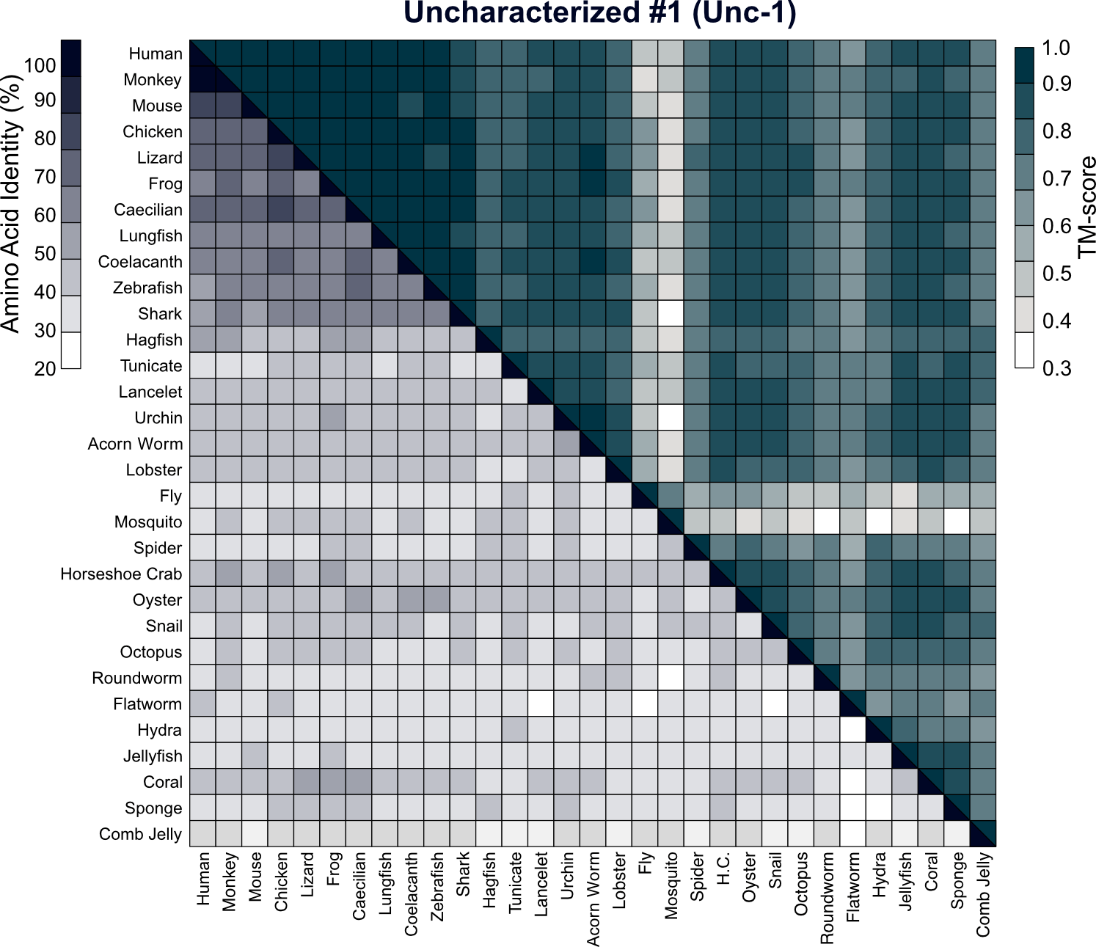


**Figure S4: Sequence-structure matrix for uncharacterized domain #1.**  Flexible alignments of the ANKLE2 Unc-1 reveal highly similar structure among evolutionary distant animals. Fly and mosquito orthologs have noticeably decreased TM-scores relative to others due to apparent insertions of disordered proteins within the region (Figure 2), which were not removed during this analysis.


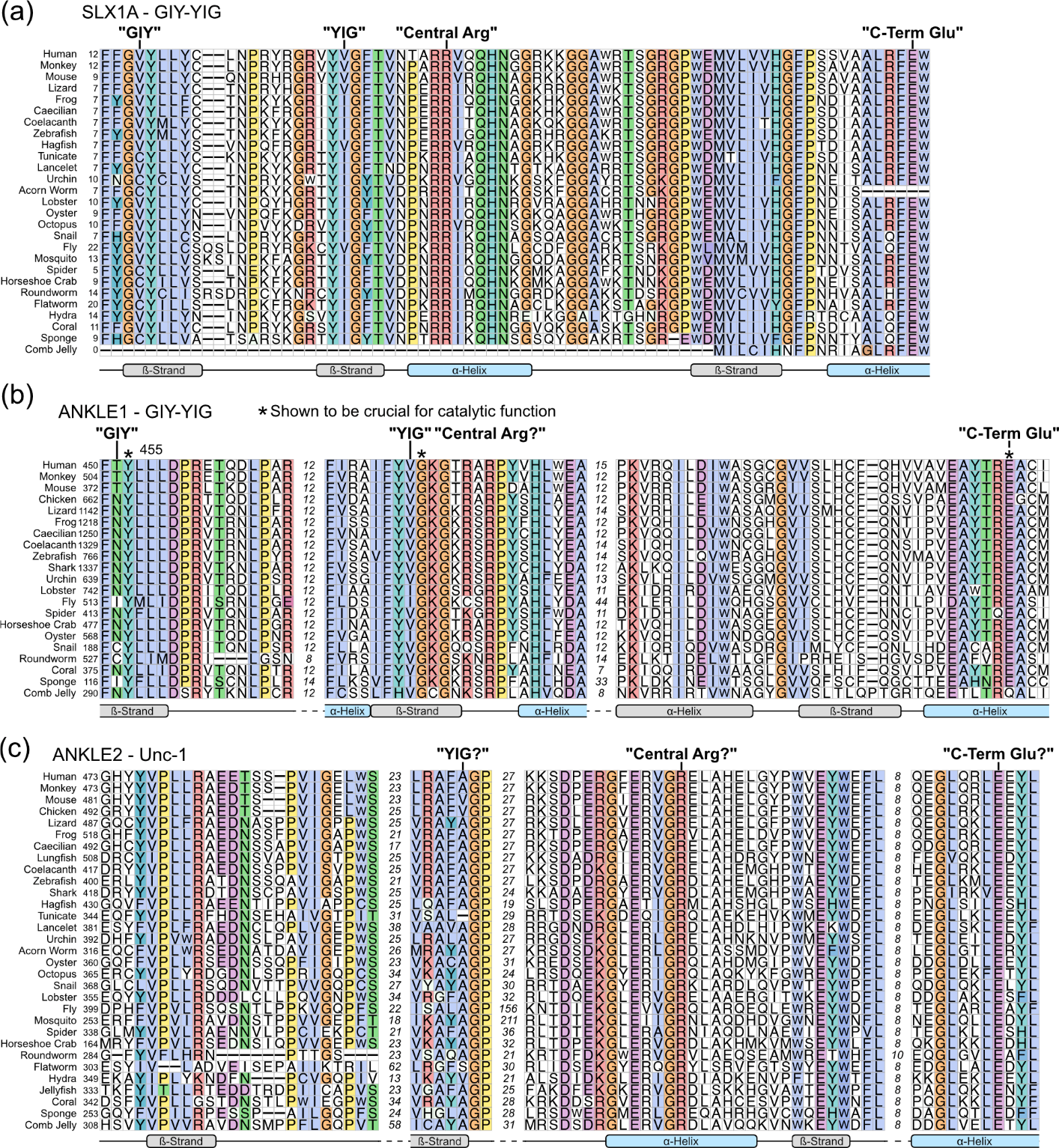


**Figure S5: Sequence alignments of GIY-YIG regions of SLX1A, ANKLE1, and ANKLE2.** (a-c) Amino acid sequences of SLX1A, ANKLE1, and ANKLE2 orthologs were acquired from NCBI and aligned using MUSCLE. Sequences were evaluated for conserved GIY-YIG motifs based on known patterns (Dunin-Horkawicz et al. 2006, Brachner et al. 2012). Certain amino acids are colored based on biochemical properties to highlight conservation.


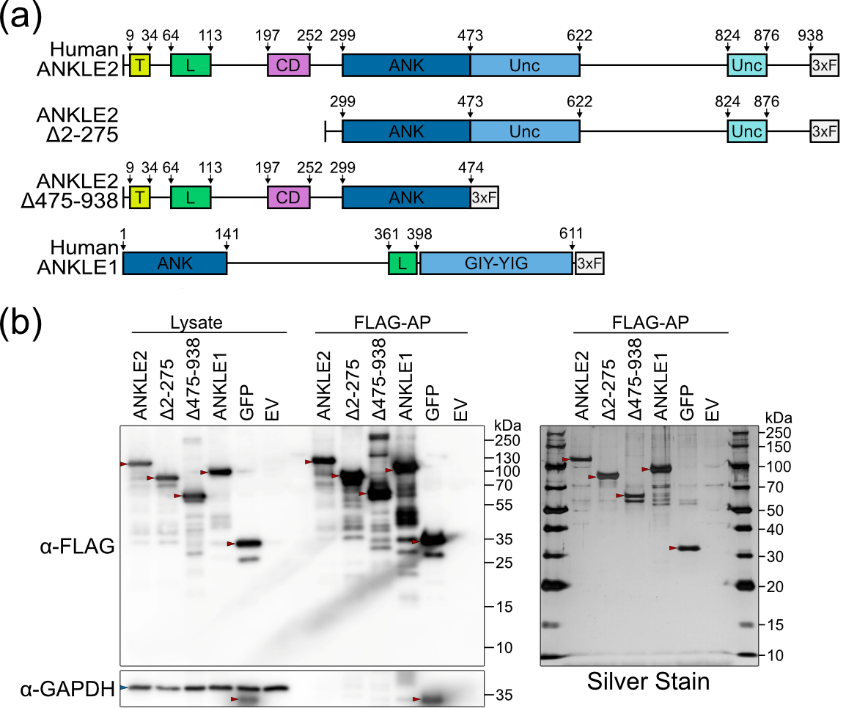


**Figure S6: Protein production and purification validation.** (a) ANKLE2-3xFLAG, truncations, or ANKLE1 were cloned into pcDNA expression vectors using Gibson assembly. (b) Proteins were expressed in HEK293T cells and purified using FLAG affinity purification (FLAG-AP). Red arrows indicate expected bands. Bands beneath each expected band represent known C-terminal degradation products and buffer composition/pH were optimized to maximize protein integrity.


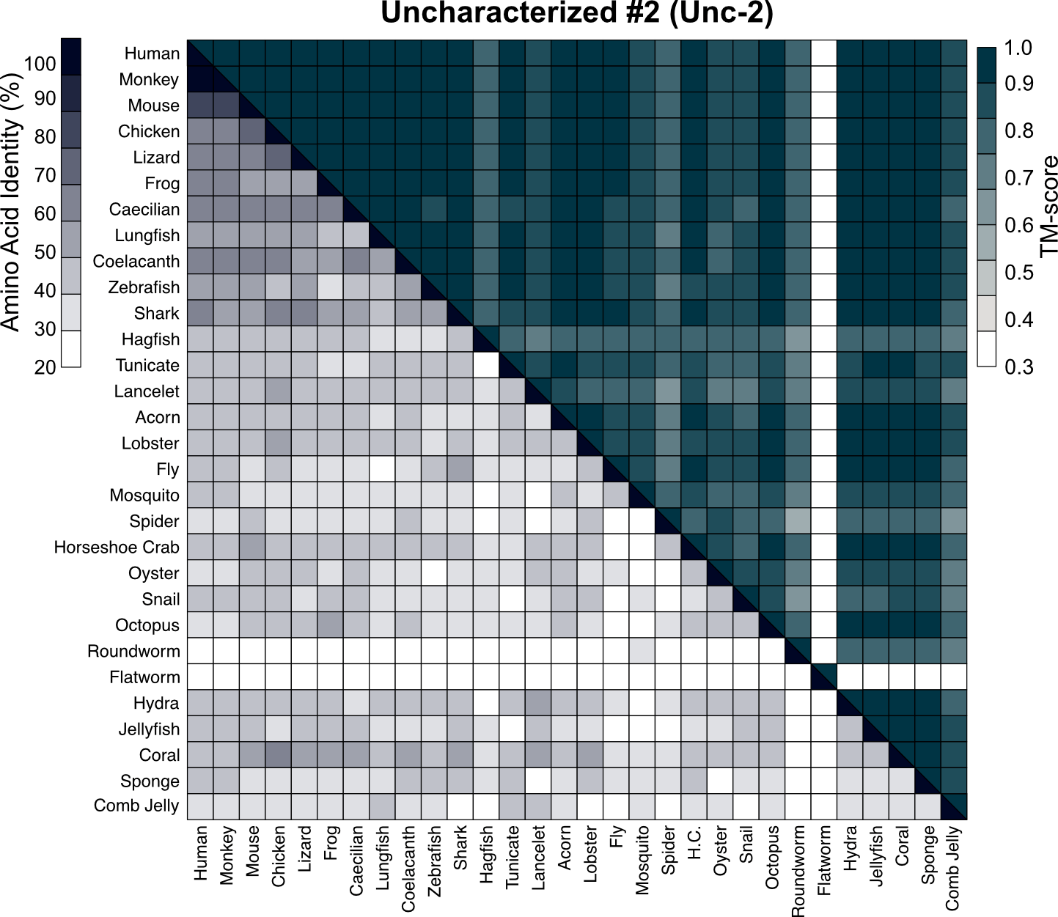


**Figure S7:** **Sequence-structure matrix for uncharacterized domain #2.** Flexible alignments of the ANKLE2 Unc-2 reveal highly similar structure among evolutionary distant animals.


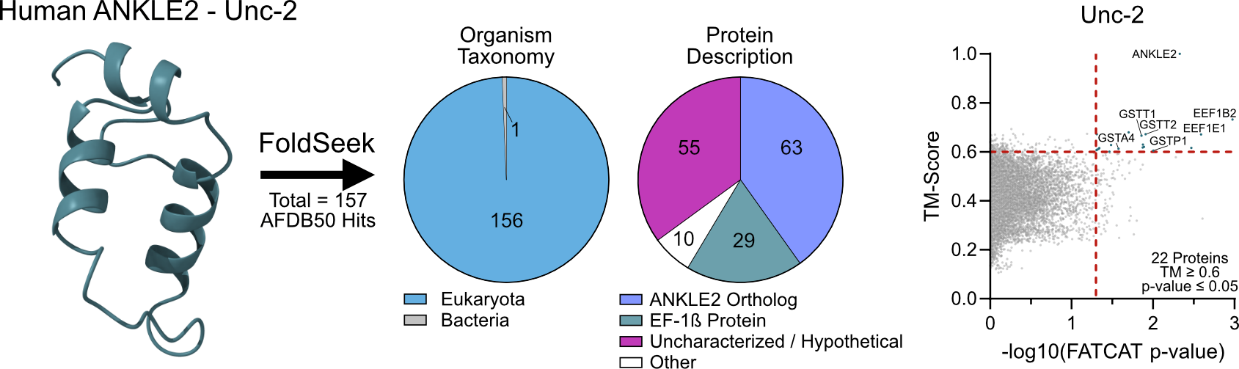


**Figure S8: Foldseek results for ANKLE2 uncharacterized domain #2.** Human ANKLE2 Unc-2 was entered into Foldseek without species restriction. Initial results reveal similarities with eukaryotic proteins. Most of the characterized hits that were not clear ANKLE2 orthologs were GST- and EF-1ß proteins. This similarity was confirmed with FATCAT🡪US-align flexible protein alignments which consistently showed GST and EF-1ß proteins among the top scores.


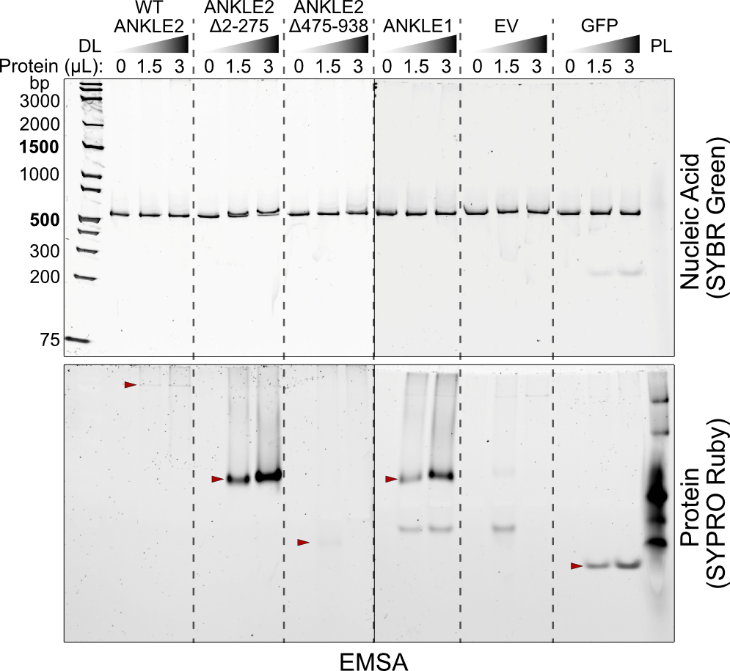


**Figure S9: ANKLE2 does not bind with dsDNA by electrophoretic mobility shift assay.** Purified proteins (Figure S6) were combined with a ~500 bp portion of non-specific DNA and allowed to bind prior to assessment by native PAGE. The gel was first stained for nucleic acids with SYBR green before staining for protein with SYPRO ruby.


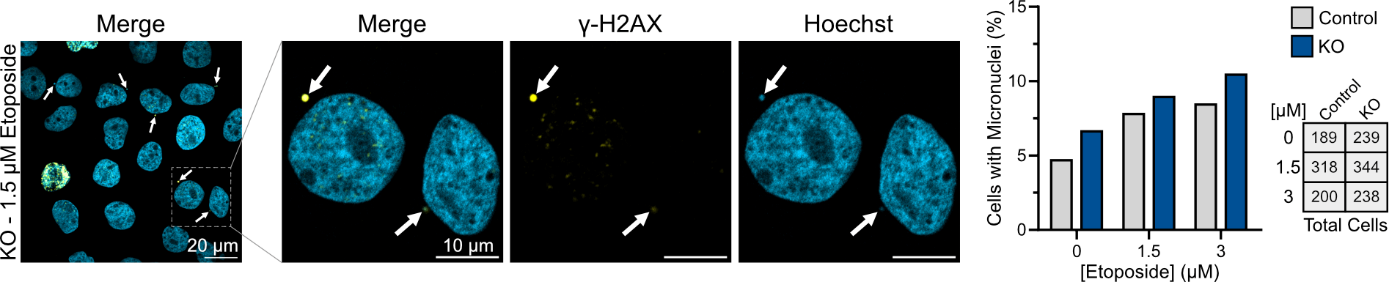


**Figure S10: Observations of micronuclei during etoposide treatment.**  Huh7 control and ANKLE2 knockout (KO) cells were treated with etoposide at noted concentrations as described in Figure 9. During quantification of γ-H2AX foci, an increase in micronuclei was also observed (white arrows). Cells were quantified across two independent experiments.

**Table S2: Sequences**

| **Organism** | **Species** | **Protein (Ortholog)** | **Accession Number** |
| --- | --- | --- | --- |
| Human | *Homo sapiens* | ANKLE2 | NP_055929.1 |
| Monkey | *Macaca mulatta* | ANKLE2 | XP_028686072.1 |
| Mouse | *Mus musculus* | Ankle2 | NP_001240743.1 |
| Chicken | *Gallus gallus* | ANKLE2 | XP_025011307.2 |
| Lizard | *Pogona vitticeps* | ANKLE2 | XP_020665561.1 |
| Frog | *Xenopus tropicalis* | ankle2 | XP_004910629.2 |
| Caecilian | *Rhinatrema bivittatum* | ANKLE2 | XP_029475159.1 |
| Lungfish | *Protopterus annectens* | ANKLE2 | XP_043916604.1 |
| Coelacanth | *Latimeria chalumnae* | ANKLE2 | XP_064424098.1 |
| Zebrafish | *Danio rerio* | ankle2 | NP_001119867.1 |
| Shark | *Carcharodon carcharias* | ankle2 | XP_041058902.1 |
| Hagfish | *Myxine glutinosa* | LOC137428901 (ANKLE2) | XP_067974861.1 |
| Tunicate | *Styela clava* | LOC120330042 (ANKLE2) | XP_039252790.1 |
| Lancelet | *Branchiostoma floridae* | LOC118409528 (ANKLE2) | XP_035666506.1 |
| Urchin | *Strongylocentrotus purpuratus* | ankle2 | XP_030837162.1 |
| Acorn worm | *Saccoglossus kowalevskii* | LOC100367770 (ANKLE2) | XP_002736187.1 |
| Lobster | *Homarus americanus* | Ankle2 | XP_042238960.1 |
| Fly | *Drosophila melanogaster* | Ankle2 | NP_001285375.1 |
| Mosquito | *Aedes aegypti* | Ankle2 | XP_021712546.1 |
| Spider | *Parasteatoda tepidariorum* | Ankle2 | XP_042900577.1 |
| Horseshoe Crab | *Limulus polyphemus* | Ankle2 | XP_022256190.1 |
| Oyster | *Saccostrea cucullata* | LOC134270048 (ANKLE2) | XP_062608216.1 |
| Octopus | *Octopus bimaculoides* | LOC106881356 (ANKLE2) | XP_052825279.1 |
| Snail | *Pomacea canaliculata* | LOC112559418 (ANKLE2) | XP_025086451.1 |
| Roundworm | *Caenorhabditis elegans* | lem-4 | NP_001023514.1 |
| Flatworm | *Schistosoma haematobium* | ANKLE2_1 | XP_051067024.1 |
| Hydra | *Hydra vulgaris* | LOC101238853 (ANKLE2) | XP_012563523.2 |
| Jellyfish | *Rhopilema esculentum* | LOC135682412 (ANKLE2) | XP_065053365.1 |
| Coral | *Stylophora pistillata* | LOC111323494 (ANKLE2) | XP_022782595.1 |
| Sponge | *Sycon ciliatum* | LOC135817761 (ANKLE2) | XP_065187074.1 |
| Comb Jelly | *Bolinopsis microptera* | LOC134811577 (ANKLE2) | XP_063674614.1 |
| Human | *Homo sapiens* | RNASEH1 | NP_002927.2 |
| Monkey | *Macaca mulatta* | RNASEH1 | NP_001244787 |
| Mouse | *Mus musculus* | RNASEH1 | NP_035405 |
| Zebrafish | *Danio rerio* | RNASEH1 | NP_001002659 |
| Human | *Homo sapiens* | ANKLE1 | NP_689576.6 |
| Hydra | *Hydra vulgaris* | ANKLE1 | CDG68700 |
| Zebrafish | *Danio rerio* | ANKLE1 | NP_999909.1 |
| Human | *Homo sapiens* | SLX1A | NP_001014999.1 |
| Bacteria | *Bacteroidota bacterium* | GIY-YIG nuclease family protein | RLD31106.1 |

**Table S3: Antibodies**

| **Antibody** | **Host Species** | **Dilution Used** | **Supplier (Catalog #)** | **RRID** |
| --- | --- | --- | --- | --- |
| GAPDH | Mouse | 1:1000 (WB) | Fisher (PIMA515738) | AB_2537652 |
| FLAG-M2 | Mouse | 1:200 (IF), 1:1000 (WB) | MilliporeSigma (F1804) | AB_262044 |
| SERCA2 | Mouse | 1:100 (IF), 1:1000 (WB) | Invitrogen (MA3919) | AB_325502 |
| Lamin A/C | Mouse | 1:1000 (WB) | Cell Signaling (4777) | AB_10545756 |
| FLAG | Rabbit | 1:400 (IF) | Cell Signaling (14793) | AB_2572291 |
| VAPA | Rabbit | WB (1:1000) | Proteintech (15275-1-AP) | AB_2256991 |
| PPP2R1A | Rabbit | WB (1:1000) | Abcam (ab154551) | N/A |
| PPP2CB | Rabbit | WB (1:1000) | Abcam (ab168371) | AB_2892220 |
| γ-H2A.X (Ser139) | Mouse | 1:250 (IF) | Sigma (05-636-I) | AB_2755003 |
| Anti-Mouse IgG-HRP | Rabbit | 1:5000 (WB) | SouthernBiotech (6170-05) | AB_2796243 |
| Anti-Rabbit IgG-HRP | Goat | 1:5000 (WB) | SouthernBiotech (4030-05) | AB_2687483 |
| Anti-Mouse AlexaFlour-488 | Goat | 1:1000 (IF) | Invitrogen (A28175) | AB_2536161 |
| Anti-Mouse AlexaFlour-555 | Goat | 1:1000 (IF) | Invitrogen (A21422) | AB_2535844 |
| Anti-Rabbit AlexaFlour-488 | Goat | 1:1000 (IF) | Invitrogen (A11008) | AB_143165 |
| Anti-Rabbit AlexaFlour-555 | Goat | 1:1000 (IF) | Invitrogen (A27039) | AB_2536100 |
